# The effect of adding real-world evidence to regulatory submissions on the breadth of population indicated for rare disease medicine treatment by the European Medicines Agency

**DOI:** 10.1186/s40545-022-00433-z

**Published:** 2022-05-04

**Authors:** Ravi Jandhyala

**Affiliations:** 1 Medialis Ltd, 13 Horse Fair, Banbury, OX16 0AH UK; 2 Centre for Pharmaceutical Medicine Research, King’s College University, London, UK

**Keywords:** Real-world evidence, Medicine adoption model, Randomised controlled trial, Orphan medicine, Multiple stakeholder approach

## Abstract

**Background:**

Despite calls for the use of additional real-world evidence (RWE) during drug development, rates of inclusion at the regulatory stage remain low. The medicine adoption model suggests that providing additional RWE to regulators would result in a wider indicated population than providing randomised-controlled trial evidence (RCTE) alone. Here, we tested this hypothesis.

**Methods:**

All engagements concerning the 88 orphan drugs approved between 2009 and 2019 on the European Medicines Agency Orphan Register were reviewed between September and December 2019. Engagements were grouped as containing either randomised-controlled trial evidence (RCTE) or RCTE with real-world evidence (RWE). The data on indicatable population (the therapeutic indication requested by an engagement) and indicated population (the therapeutic indication ultimately granted) as well as the median number of criteria limiting the indicated population in each study type (RCTE/RWE) was extracted. A chi-square test assessed the association between the indicated population (as a proportion of the indicatable population) and type of evidence (RCTE with or without RWE) and a Wilcoxon rank sum test assessed the difference between the median number of limiting criteria between RCTE and RWE studies. Prediction modelling extrapolated the results of a power analysis to a level expected to deliver significance and the time this would take.

**Results:**

The review identified 103 engagements, of which three were excluded (one contained only RWE; two contained only systematic literature reviews), leaving 100 engagements for 87 orphan medicines in the final analysis. Only 13% of engagements contained RWE. Although the difference was statistically insignificant, 76.92% of engagements containing RCTE and RWE resulted in a broader indicated population as compared to only 56.32% of those that contained RCTE alone. The median number of limiting criteria from RCTE (37 (28, 43)) and RWE (5 (2, 9)) studies varied significantly (*p* = 0.005). Modelling suggested that the analysis would achieve sufficient power by 2033–37 at the current RWE adoption rate.

**Conclusion:**

The proportion of the disease population studied in RWE was greater than that in RCTE. The analysis testing the relationship between additional RWE and broader indicated population would achieve adequate power between 2032 and 2037 at the current RWE adoption rate.

## Background

In 2020, the European Medicines Agency (EMA) published EMA Regulatory Science to 2025, which included among its top five core recommendations the promotion of the use of high-quality real-world data in regulatory decision making [1]. The OPTIMAL framework is a set of criteria developed by the EMA for the regulatory use of RWE, which addresses its operational, technical and methodological challenges [2]. Although there is recognition of the need to include RWE in regulatory submissions, and efforts have been made to define the bounds of its appropriate use, the development of firm guidelines remains a strategic priority [1, 2]. As such, RWE is not yet widely provided alongside clinical trials in regulatory submissions, with its inclusion commonly limited to post-approval research and safety monitoring [3, 4]. However, the recent evidence proposing the medicine adoption model has suggested that the inclusion of RWE at the regulatory stage may increase the depth of maximal adoption by 31 patients for every 100 trial patients and decrease the time to maximal adoption by 22 months for every 100 trial months [5]. This shows the potential for profound benefits resulting from the early generation of RWE alongside clinical trials for both pharma and patients.

Based on the systems theory [6], the medicine adoption model has suggested that the approval, reimbursement, prescription and receipt of medicines occurs in an open system comprising three sequential subsystems involving regulators, payors and prescribers [5]. Each subsystem requires a specific set of evidence upon which to base their decisions. This is normally provided in the form of randomised-controlled trial evidence (RCTE) and stakeholder-specific RWE. This evidence is appraised by each subsystem, and if its internal logic is satisfied, and the output is favourable, then the medicine undergoing approval progresses to the next system. In the last subsystem, the prescriber approves the decision to treat patients with the medicine, at which point, patients receive it. Thus, for medicines to reach patients through this system, the evidence provided to gatekeeper stakeholders must answer the research questions asked by each subsystem.

The three subsystems can be considered heterogeneous in their attitudes to RWE and therefore require a multiple stakeholder approach to RWE generation [7] to ensure the timely progression of a medicine to appropriate patients. Put simply, a reliance on RCTE alone at any stage of adoption is likely to be suboptimal. In addition, this research suggested that the prescriber controls the time to maximal adoption of a drug and that the expected increase in the depth of maximal adoption was due to the propensity of RWE to represent a greater proportion (breadth) of the disease population [7]. Failing to utilise RWE to its greatest benefit at the time of regulatory submission may, therefore, prevent an indicatable population from receiving a drug from which it may benefit. The proportion of the disease population indicated for treatment with a new medicine is defined by the regulator subsystem, which has traditionally been supplied with RCTE alone, despite its well-documented limitations regarding its generalisability to the real world. The additional influence of RWE on this subsystem and its output of indicated proportion of a disease population is the subject of this research.

## Methods

### Study aims

**Fig. 1:**
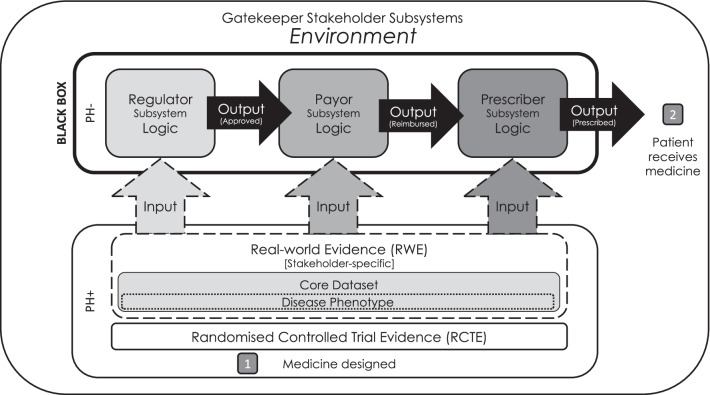
The medicine adoption model: PH − (non-pharmaceutical company stakeholder groups); PH + (pharmaceutical company stakeholder groups)

The primary study aim was to determine whether the addition of RWE to RCTE during regulatory engagements for clinical trials increased the breadth of the population indicated to receive rare drug medications as compared to that indicated by regulatory engagements during which RCTE was submitted alone. We expected a directly proportional effect between the sizes of the respective indicated populations. If we were unable to determine this, then we wanted to know when this effect would likely be detectable given the current rate of increase in the addition of RWE to RCTE during regulatory engagements. The secondary aim was to determine whether the indicatable population in RWE submitted during regulatory engagements was broader than that in the RCTE it accompanied to determine the underlying mechanism for any possible effect (Fig. 1).

### Design, materials and processes

**Fig. 2:**
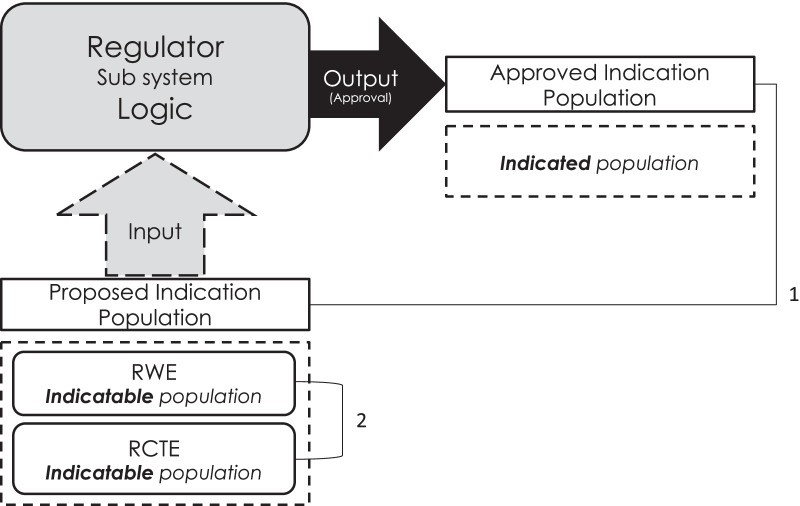
Schematic diagram of the analyses conducted to answer the research question

This research was an analysis of data concerning all orphan drugs approved between 2009 and 2019 on the European Medicines Agency (EMA) Orphan Register. Between September and December 2019, a review of the specified engagements was conducted. An engagement comprised an individual submission of evidence supporting a medicine by the marketing authorisation applicant/holder (MAA/H) to the EMA and the EMA’s subsequent response. We categorised the constituent evidence as either RWE or RCTE. Then, we determined the size of indicatable population (the therapeutic indication requested by an engagement) and indicated population (the therapeutic indication ultimately granted) for each type of evidence by measuring the frequency count of criteria limiting the types of patients who could receive a drug. For example, the indicatable population in the first engagement for Adcetris included patients with relapsed or refractory Hodgkin lymphoma and those with relapsed or refractory systemic anaplastic large cell lymphoma (criteria count = 4). It was assumed that more limiting criteria represented a narrower indicatable/indicated population. The indicatable population included limiters extracted from both RCTE and RWE when available (Fig. 2). Engagements were excluded if they contained only RWE, if they contained neither RCTE nor RWE or if information on proposed or indicated populations was unavailable. Each engagement was considered conditionally independent for drugs with more than one engagement due to the supporting evidence related to a separate proposed population.

### Statistical analyses

A bar plot visualised the distribution of engagements and types of evidence they contained. A chi-square test was conducted to assess the association between the indicated population and the type of evidence (RCTE with or without RWE). A Wilcoxon’s rank sum test assessed the difference in the frequency count of limiting criteria between RCTE and RWE studies. For all analyses, *p* values ≤ 0.05 were considered statistically significant, and R version 4.0.2 was used. A power analysis evaluated the influence of the small sample size of RWE studies on the overall results. Prediction modelling extrapolated these results to a level expected to deliver significance and the time this would take. The modelling based on its accrual rates of future studies (or engagements) on the increase of studies from 2010–2014 to 2015–2019 and assumed the rise in RCT studies was linear. The model considered the increase in future RWE in both linear and exponential settings. The results presented the power required to detect the observed difference in the indicated population between the RCTE and RCTE + RWE groups as constant.

## Results

**Fig. 3:**
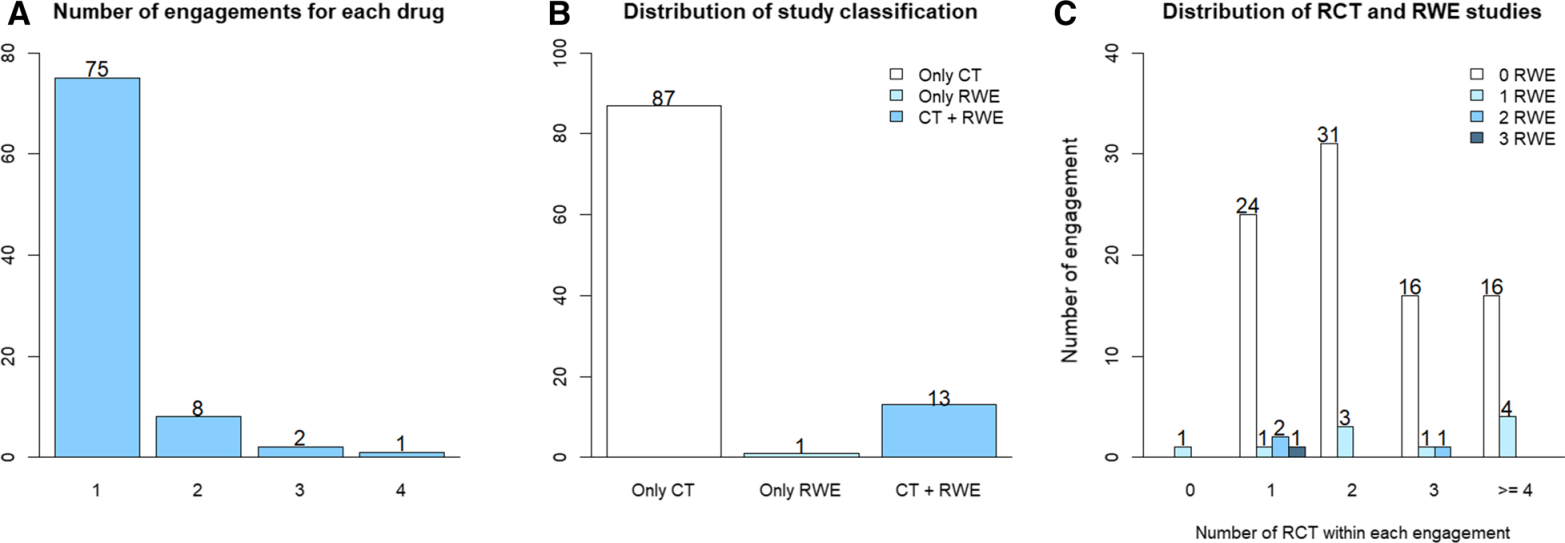
Data summary: **A** Distribution of engagements for each drug, **B** Distribution of study types within engagements, and **C** Distribution of RCT and RWE studies

The review identified 103 engagements in total, of which three were excluded (one contained only RWE; two contained only systematic literature reviews), leaving 100 engagements for 87 different orphan medicines in the final analysis. Of the 87 medicines included, 11 had more than one engagement. Of the 100 engagements included, 87 consisted of only RCTE, 13 consisted of RWE and RCTE, and one consisted of only RWE. Figure 3 provides the distribution of number of engagements for each drug, types of study included in engagements and the overall distribution of study types. Table 1 provides the difference in indicated population for each type of evidence included in engagements (RCTE with or without RWE). The findings showed a broader indicated population in 49 of 87 engagements (56.32%) in which RCTE was submitted alone compared to 10 of 13 engagements (76.92%) in which RCTE was submitted alongside RWE. However, this observed difference was not statistically significant (*p* = 0.269).

**Table 1:** Difference in indicated population by type of evidence included in engagements

Evidence profile	Approved vs. proposed indication populations			*p*-value
	Same or narrower	Broader	Total	
RCTE	38 (43.68%)	49 (56.32%)	87	0.269
RCTE + RWE	3 (23.08%)	10 (76.92%)	13	
Total	41	59	100	

**Fig. 4:**
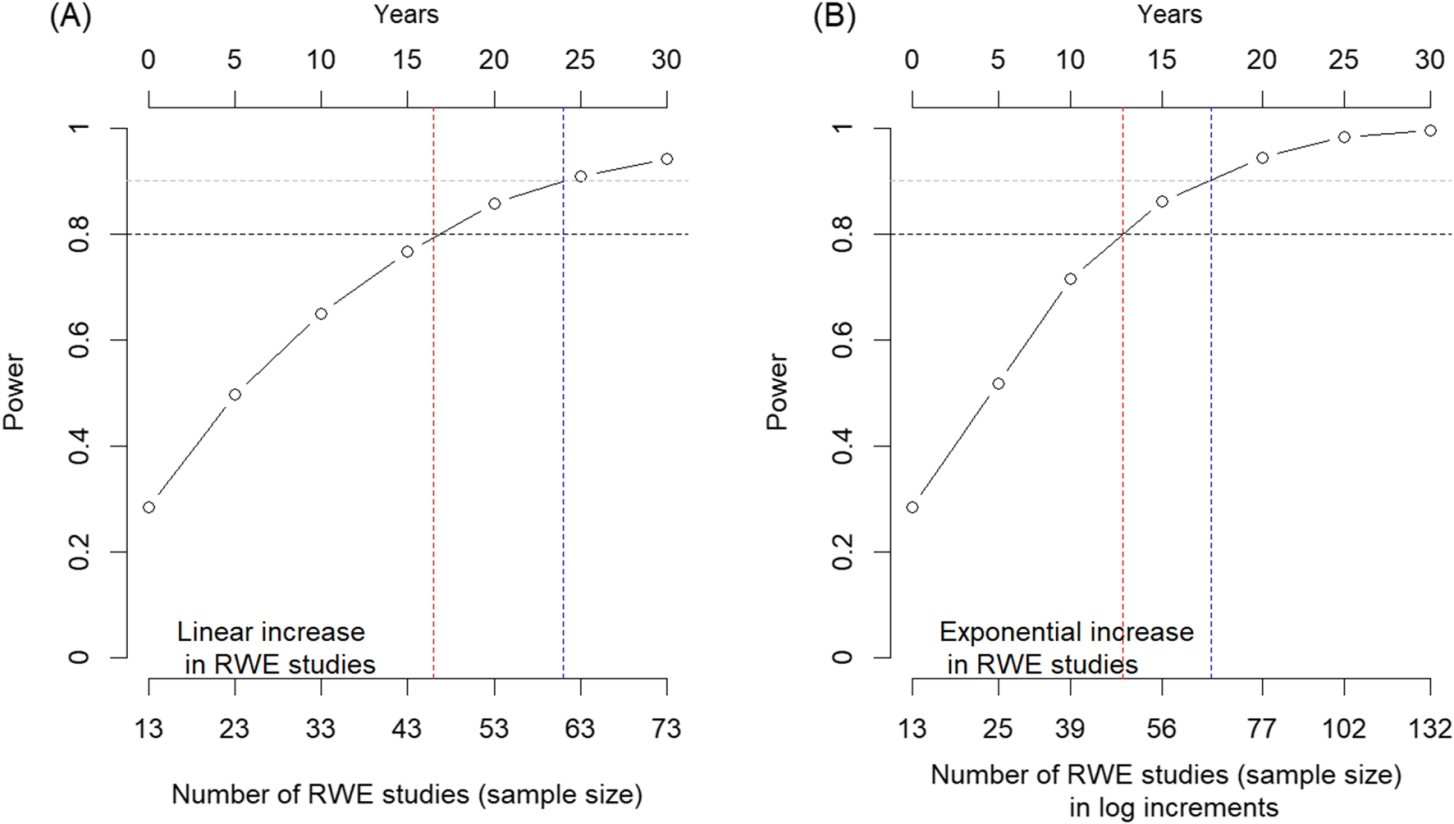
Power analyses based on the predicted number of studies: panel **A** linear increase in RWE studies; panel **B** exponential increase in RWE studies. Time 0 is 2019. The dashed red line denotes 80% power; dashed blue line indicates a power of 90%

As shown in Table 2, the median number of unique limiters extracted from RCTE was 37 (28, 43) and from RWE, it was 5 (2, 9). This difference was statistically significant (*p* = 0.005). A power analyses based on the number of studies estimated to be submitted during regulatory engagements over the course of the next 30 years showed that a power of 80% to detect a 20% difference (as observed during 2009–2019) between the two groups (RCTE with and without RWE) will be achieved in around 17 years (~ 2037) with a linear increase and in approximately 13 years (~ 2033) with an exponential rise (Fig. 4).

**Table 2:** Total number of unique limiters by evidence type for engagements with RCTE and RWE

Evidence type	Total limiter count	
	Median (IQR: Q1, Q3)	*p* value
RCTE	37 (28, 43)	0.005
RWE	5 (2, 9)

## Discussion

The primary research question sought to test the hypothesis carried forward from previous work, which aimed to determine whether the addition of RWE to RCTE would increase the population indicated for a new medicine. Although the small number of engagements involving RWE limited the power of this analysis, there was a trend toward a broader indicated population for engagements including RCTE and RWE (76.92%) compared to those in which RCTE was submitted alone (56.32%). However, this difference was not statistically significant (*p* = 0.269). Predictive modelling showed that sufficient power to detect a significant difference between these two groups would be achieved in 13 to 17 years depending on whether exponential or linear growth in the inclusion of RWE in regulatory engagements occurs. When both RCTE and RWE were submitted during regulatory engagements, the population indicated by RWE was significantly broader than that indicated by the RCTE it accompanied. This supported the hypothesis generated from the previous work on the breadth of indicated population, even if the resultant effect size is still emerging.

Engagements that contained RWE comprised a relatively small percentage (13%) of the 100 engagements included in the analysis. This was unsurprising, as regulatory authorities are currently still in the process of encouraging pharmaceutical companies to generate and include RWE in submissions for marketing authorisation and developing firm guidelines for this [1]. Currently, barriers to the design and implementation of RWE generation during drug development prevent its synchronisation with regulatory submission. For example, clinical development teams tend to have a short-term perspective due to their traditional focus on the regulatory subsystem and securing regulatory approval to the exclusion of other subsystems and gatekeeping tasks. Downstream objectives may be considered the responsibility of post-marketing functions, such as commercial, market access and medical affairs. To synchronise RWE and RCTE delivery and optimise the adoption of a new medicine, RWE must be initiated at the same time as Phase III studies [8].

The likelihood of regulators approving a broader indication with the inclusion of RWE in an engagement may be explained by previous research on the medicine adoption model, which suggested that the prescriber controls the depth of maximum adoption. This is because the addition of RWE to RCTE during regulatory engagements would increase the likelihood of approval and shorten the time taken by the prescriber to prescribe the approved medication to a patient. As the initial subsystem in the model, control of the population able to receive the medicine lies with the regulator, because reimbursement decisions are mainly limited to the indicated population the regulatory subsystem approves. A possible exception is the prescriber subsystem, which has the flexibility to prescribe a medicine outside the indicated population if suitably justified. This argument aligns with existing evidence, suggesting that evidence from RCTE alone may not be sufficient to establish the cost-effectiveness of new technologies [9]. A fundamental prerequisite for adopting a new medicine is evidence of a favourable risk–benefit profile in a specific patient population. We hypothesised that additional RWE would increase the breadth of the disease population showing this favourable risk–benefit and in doing so, satisfying the internal logic of the regulatory subsystem, enabling a broader indicated population. Early RWE delivery operationalisation still faces challenges beyond engaging downstream functions at the Phase III design phase [10].

Understanding the vehicle to use in generating RWE is an immediate priority [11]. Patient registries or observational cohort clinical studies of a prospective and or retrospective type are arguably the only choices available to conduct the primary search [12, 13]. Issues still exist around the nature of the real-world data collected, notably missing data from that requested and how well that which is collected accurately reflects the disease phenotype [11, 12]. A recently described theory on construct measurement advocated observing any recruit into a clinical study with Neutrality to ensure all effects are recognised [13]. Examples of how failing to apply Neutral Theory when measuring health-related quality of life have been documented [13]. To apply these principles, investigators must take care to research and develop a core dataset that contains all relevant indicators with the exclusion of all irrelevant ones. The core dataset and the need to engage with patients in the development of patient registries in the rare disease setting were two quality indicators unanimously agreed upon by a multidisciplinary panel of experts [14].

These core indicators also need to be tested as falling within standard diagnostic and monitoring practice to avoid the study being considered a clinical trial by certain regulatory authorities [15]. Given the documented heterogeneity in research questions across the gatekeeper stakeholders in the medicine adoption model, their engagement with any core dataset development is essential for ensuring the planned study can answer their research questions. The findings will then constitute ‘evidence’ from their perspectives [14]. Thus, a neutral list of indicators in a real-world study can only be achieved through exhaustive targeted research with each gatekeeper stakeholder. It follows that the optimal real-world study design will consider both the medicine’s adoption model and the multiple stakeholder approach to RWE generation. Further work, beyond indicator development, will involve researching the logic within each of these subsystems to inform the design of RWE, ensuring it is not only delivered at the earliest opportunity, but is on target and fit for purpose in facilitating the adoption of a new medicine [16].

In the context of rare disease medicine, patients’ registries are a valuable source of RWD [17]. To strengthen the contribution of RWE to the regulatory submission, MAA/Hs must take all reasonable steps to include every patient receiving a medicine outside the RCT in a patient registry. An approach could entail enrolling such patients accessing treatment via early access schemes, compassionate use programs [18] and named patient requests. Although organisational aspects of the inclusion of RWE at the regulatory stage have been less well-studied, initiating its generation in conjunction with early access schemes could exploit lead times with minimal effect on overall duration of regulatory engagement [19]. With the exclusion of heterogenous groups with multiple comorbidities from rare disease RCTs to ensure the internal validity of findings, RWE generated from such groups could be a complementary addition during the regulatory stage [19]. However, selection bias and type I errors arising from small, non-representative cohorts in RWE are risks that must be addressed when determining the suitability of RWE for regulatory use and the use of statistical methods to address patient population differences should be considered [20]. Finally, it is essential to provide the appropriate context for any research for the significance of the findings to be fully appreciated. In this case, proper planning and design of RWE and its synchronised delivery with RCTE in time for regulatory submission can not only maximise the number of patients who receive a new medicine sooner but also, more importantly, patients who would otherwise have been left untreated or on suboptimal treatment now have an opportunity to be included in the indicated population. Therefore, more patients benefit sooner. Every patient deserves optimal treatment and this can be achieved by delivering optimised evidence to decision-makers at the earliest opportunity.

### Limitations

The scope of this study was limited to orphan medicines in Europe. Although there may not be firm evidence that this necessarily limits the generalisability of the findings to North America or other regions, such as China and Japan, it should be considered a limitation. The medicine adoption model is unlikely to differ between rare and non-rare medicines, but in the absence of a firm confirmation, this needs to be recognised as a potential limitation.

### Future directions

Recognising that the primary research question for this study remains unanswered, as a result of limited engagements containing RWE, a critical next step would be to repeat this exercise at a future date. In terms of elaborating on the logic being employed in the regulatory subsystem, some information now exists. However, further research is required to generate a neutral list of rules and principles used by the regulator subsystem, the payor and the prescriber.

## Conclusions

Although increasing, the amount of RWE delivered alongside RCTE as part of regulatory submissions is currently insufficient to achieve the appropriate statistical power to know whether it increases the breadth of indicated or approved population. The answer will be available between 13 and 17 years from now at the current rate of submission. As a proportion of the overall disease population, the populations observed in RWE are significantly larger than those in RCTE; thereby, providing a mechanistic basis for the emerging effect on the size of the population that the regulatory subsystem approves the new medicine to be used in.

## Data Availability

The datasets used and/or analysed during the current study are available from the corresponding author on reasonable request.

## Abbreviations

RWE: Real-world evidence
RCTE: Randomised-controlled trial evidence
EMA: European Medicines Agency
MAA/H: Marketing authorisation applicant/holder
